# Effect of intraoperative fluid volume on postoperative ileus after robot-assisted radical cystectomy

**DOI:** 10.1038/s41598-021-89806-z

**Published:** 2021-05-18

**Authors:** Ji Sung Shim, Tae Il Noh, Ja Hyeon Ku, Sangchul Lee, Tae Gyun Kwon, Tae-Hwan Kim, Seung Hyun Jeon, Sang Hyup Lee, Jong Kil Nam, Wan Seok Kim, Byong Chang Jeong, Ji Youl Lee, Sung Hoo Hong, Koon Ho Rha, Woong Kyu Han, Won Sik Ham, Young Goo Lee, Yong Seong Lee, Sung Yul Park, Young Eun Yoon, Sung Gu Kang, Jong Jin Oh, Seok Ho Kang, Korean Robot Assisted Radical Cystectomy (KORARC) Study Group, Ji Sung Shim, Tae Il Noh, Ja Hyeon Ku, Sangchul Lee, Tae Gyun Kwon, Tae-Hwan Kim, Seung Hyun Jeon, Sang Hyup Lee, Jong Kil Nam, Wan Seok Kim, Byong Chang Jeong, Ji Youl Lee, Sung Hoo Hong, Koon Ho Rha, Woong Kyu Han, Won Sik Ham, Young Goo Lee, Yong Seong Lee, Sung Yul Park, Young Eun Yoon, Sung Gu Kang, Jong Jin Oh, Seok Ho Kang

**Affiliations:** 1 Department of Urology, Korea University College of Medicine, 73 Inchon-ro, Seongbuk-gu, Seoul, 02841 Korea; 2 Department of Urology, Seoul National University College of Medicine, Seoul, Korea; 3 Department of Urology, Kyungpook National University School of Medicine, Daegu, Korea; 4 Department of Urology, Kyung Hee University School of Medicine, Seoul, Korea; 5 Department of Urology, Pusan National University Yangsan Hospital, Yangsan, Korea; 6 Department of Urology, College of Medicine, Busan Paik Hospital, Inje University, Busan, Korea; 7 Department of Urology, Samsung Medical Center, Sungkyunkwan University School of Medicine, Seoul, Korea; 8 Department of Urology, College of Medicine, Seoul St. Mary’s Hospital, The Catholic University of Korea, Seoul, Korea; 9 Department of Urology, Severance Hospital, Yonsei University College of Medicine, Seoul, Korea; 10 Department of Urology, Hallym University Kangnam Sacred Heart Hospital, Hallym University School of Medicine, Seoul, Korea; 11 Department of Urology, Hallym University Sacred Heart Hospital, Hallym University College of Medicine, Anyang, Korea; 12 Department of Urology, Hanyang University College of Medicine, Seoul, Korea; 13 Department of Urology, Seoul National University Bundang Hospital, Seongnam, Korea

**Keywords:** Urological cancer, Bladder cancer, Bladder

## Abstract

This study aimed to investigate the effect of intraoperative fluid volume on the postoperative ileus (POI) recovery period. A retrospective review of the Korean robot-assisted radical cystectomy database identified 718 patients who underwent robot-assisted radical cystectomy (RARC). Regression analyses were performed to identify the associations between the amount of intraoperative fluid administration (crystalloid/colloid/total), POI period (time to flatus/bowel movements), and length of hospital stay (LOS) after adjusting for covariates. In addition, we analyzed the risk factors for gastrointestinal complications and prolonged POI using a logistic regression model. An increasing volume of the administered crystalloid/total fluid was associated with prolonged POI (crystalloid R^2^ = 0.0725 and P < 0.0001; total amount R^2^ = 0.0812 and P < 0.0001), and the total fluid volume was positively associated with the LOS (R^2^ = 0.099 and P < 0.0001). The crystalloid amount was a risk factor for prolonged POI (P < 0.001; odds ratio, 1.361; 95% confidence interval, 1.133–1.641; P < 0.001). In the context of RARC, increased intravenous fluids are associated with prolonged POI and longer LOS.

## Introduction

Since its introduction in 2003, robot-assisted radical cystectomy (RARC) is a minimally invasive technique that has been increasingly used worldwide. It shows comparable results in terms of oncologic and perioperative outcomes^1,2^. Recent studies have shown that RARC improves perioperative morbidity and facilitates early recovery^3,4^.

Gastrointestinal complications are the primary complications of both RARC and open surgical approach. Postoperative ileus (POI), which occurs in approximately 20% of patients, is commonly implicated for delayed recovery and increased risk of postoperative complications^5^. Weak and ineffective peristalsis associated with POI can lead to accumulation of gastrointestinal secretion, causing abdominal distension and vomiting; prolonged POI even requires parenteral nutrition^6^. POI is traditionally considered inevitable after major abdominal surgery, leading to increased patient morbidity, hospital costs^7,8^, and 30-day readmission rates^9^.

A complex interplay between neurogenic, inflammatory, fluid and electrolyte, and pharmacologic components plays a role in the development of POI. Among these, fluid and electrolyte imbalances can be managed with intraoperative care. However, proper perioperative fluid management strategies for patients undergoing elective surgery have not yet been established^10–13^. Several studies have reported that hypervolemic management may result in bowel edema, prolong the recovery of bowel function, disrupt tissue oxygenation, and adversely affect the healing of anastomotic sites^14–17^. On the other hand, fluid restriction has been shown to accelerate the recovery of bowel function and facilitate the early intake of oral diet; but owing to the heterogeneity of these studies and the lack of a precise definition of “restricted,” the real impact of fluid restriction on the POI period is yet to be established^18,19^. In an attempt to overcome this limitation, we attempted to determine the independent effect of the intraoperative volume on the POI period and length of hospital stay (LOS) through regression analysis, after adjusting for other covariates.

The Korean Robot-Assisted Radical Cystectomy (KORARC) study group has a quality assurance database comprising > 750 RARC patients. In this study, the KORARC database was queried for the oncologic and functional outcomes of a large series of patients who underwent RARC. Using this database, we tested the hypothesis that unrestricted fluid management in patients during operation could be associated with prolonged bowel function convalescence.

## Materials and methods

### Study population

The prospectively collected KORARC database comprises over 750 patients from 11 tertiary referral institutions (21 surgeons) who were treated with RARC between April 2007 and October 2019. Study participants included patients with recurrent/multiple or high-grade superficial or muscle-invasive bladder cancer without evidence of metastatic disease on preoperative imaging. Further, eligible patients medically cleared for radical cystectomy with pelvic lymph node dissection (PLND), were aged ≥ 18 years, and had clinical stage Ta–T4/N0–N3/M0. We excluded study participants with evidence of metastasis who had previously undergone abdominal surgery or pelvic radiation therapy. The KORARC database is a web-based electronic database and is a brain-child of the Korean Society of Endourology and Robotics. All 718 patients enrolled in this study received perioperative patient care according to the previously established enhanced recovery after surgery (ERAS) program^20–22^. However, we adopted a slightly modified ERAS program based on special circumstances in Korea. As per anesthesiologists’ requirements, all patients fasted from 12 AM of the day preceding the surgery; a policy that applies to almost all hospitals in South Korea. Further, we could not administer alvimopan, a μ-receptor opioid antagonist, that has been reported to be effective in bowel function recovery in several studies, because it has not been introduced in Korea yet. Most of the other guidelines were followed, such as the avoidance of mechanical bowel preparation, avoidance of nasogastric tube insertion or its early removal, early ambulation, early feeding (within 24 h post-surgery), and use of multimodal analgesia to minimize opioid use.

### Definitions

Based on a systematic review, the definition of POI was proposed as follows: “POI” is defined as the bowel dysfunction that occurs immediately after surgery, and recovery of POI refers to a case in which flatus or stool is passed and oral diet is tolerated; “prolonged POI” can be defined as a case in which one or more of following conditions are applicable on or before postoperative day 7 without POI resolution as per the above-mentioned criteria: (1) nausea or vomiting, (2) inability to tolerate oral diet for the preceding 24 h; (3) no flatus or stool passage for the preceding 24 h (4) abdominal distension, and/or (5) radiologic bowel distension sign with no mechanical obstruction^23^.

### Endpoints

The primary endpoint was the correlation between the intravenous fluid volume (crystalloid/colloid/total), POI period, and LOS, after controlling for confounders. Regression analyses were performed to identify the combination of crystalloid volume, colloid volume, total fluid volume, and LOS associated with prolonged POI. The secondary endpoint was identification of gastrointestinal complications and prolonged POI. Multivariate logistic regression was used to assess the following potential predictors: age, sex, body surface area, body mass index, corporeal method (intracorporeal or extracorporeal), urinary diversion type (orthotopic bladder substitution or ileal conduit), crystalloid/colloid injection amount, operation time, American Society of Anesthesiologists (ASA) score, PLND extent, and tumor-node-metastasis (TNM) stage.

### Statistical analyses

Continuous variables are expressed as median (interquartile range [IQR]) or as a number (percentage) of cases. Categorical variables are reported as numbers and percentages. Pearson’s correlation coefficients were used for statistical comparisons of continuous variables. Logistic regression analysis was performed to analyze the predictors of complications. Simple and multiple linear regression analyses were performed. All analyses were performed using the Statistical Package for the Social Sciences (SPSS) version 20.0 (IBM Corp., Armonk, N.Y., USA) and SigmaPlot (version 13.0; SYSTAT Inc., San Jose, CA, USA). Results were considered statistically significant if the P-value was less than 0.05.

### Ethics statement

This study conformed to the standards of the Declaration of Helsinki and the current ethical guidelines. The study protocol was reviewed and approved by the Institutional Review Board of Korea University Hospital (No. 2019AN0102). Written informed consent was obtained from all the study participants prior to their enrolment.

## Results

### Baseline characteristics

The average age of patients was 65 years (IQR, 60.0–73.0), of which 615 (85.0%) were men; 57 (7.9%) had an ASA score > 3 (Table 1). As for the urinary diversion type, ileal conduit constituted 39.0% of cases; orthotopic bladder substitution, 55.0%; and continent cutaneous urinary diversion, 6.0%. The distribution of corporeal methods was as follows: intracorporeal, 15.3% and extracorporeal, 84.7%. The total number of patients who underwent standard PLND and extended type PLND was 151 (21.1%) and 397 (55.2%), respectively. Regarding perioperative outcomes, the median operation time was 433 min (IQR, 330–520), and the median time to flatus and bowel movement was 72 (IQR, 47–90) and 109 (IQR, 84–138) hours, respectively. The median LOS was 16 days (IQR, 13–22).

**Table 1:** Baseline characteristics of patients and perioperative parameters.

Baseline characteristics	Values n = 718	IQR
Age, years	65	60.0–73.0
**Sex**		
Male	610 (85.0)	
Female	108 (15.0)	
BMI, kg/m^2^	24.1	22.2–26.2
BSA	1.8	1.6–1.9
ASA score ≥ 3	57 (7.9)	
**Type of urinary diversion**		
Ileal conduit	280 (39.0)	
OBS	395 (55.0)	
CCUD	43 (6.0)	
**Diversion technique**		
Extracorporeal	536 (84.7)	
Intracorporeal	182 (15.3)	
**Types of PLND**		
Extended PLND	397 (55.2)	
Standard PLND	151 (21.1)	
Limited PLND	21 (2.9)	
None	149 (20.8)	
**Perioperative outcomes**		
Operation time (min)	433	330–520
Time to flatus (h)	72	47–90
Time to bowel movement (h)	109	84–138
Length of hospital stay (days)	16	13–22

Values are presented as number (%) or median.

*ASA* American Society of Anesthesiologists, *BMI* body mass index, *BSA* body surface area, *CCUD* continent cutaneous urinary diversion, *IQR* interquartile range, *OBS* orthotopic bladder substitution, *PLND* pelvic lymph node dissection.

### Intraoperative intravenous fluids and preoperative laboratory results

The volumes of the crystalloid, colloid, and blood products administered are shown in Table 2. The overall median volumes of intravenous fluid used were as follows: total volume, 2850 mL (IQR, 2100–4012); crystalloid, 2600 mL (IQR, 1800–3500); and colloid, 390 mL (IQR, 0–500 mL). All patients on crystalloid fluids received 1450 mL of lactated ringer’s solution (IQR, 900–2490). Among the colloid-administered patients, 308 patients received 700 mL of synthetic colloids (IQR, 500–900).

**Table 2:** Intraoperative intravenous fluids and primary preoperative laboratory results.

Baseline characteristics	Values	Injected patient no. n = 718	IQR
Total volume of intravenous fluid, mL	2850		2100–4012
Total volume of crystalloid, mL	2600		1850–3500
Total volume of colloid, mL	390		0–500
**Crystalloid, mL**			
Normal saline	1000	261	575–1700
Lactated Ringer’s solution	1450	446	900–2490
Plasma solution	1700	456	1000–2500
5%/10% dextrose solution	150	15	50–200
0.45% NaCl	600	1	–
0.9% NaCl	100	2	–
**Colloid, mL**			
5% albumin	250	18	250–450
Synthetic colloids	700	308	500–900
**Blood products, mL**			
Packed red blood cells	500	111	320–655
Fresh frozen plasma	360	6	260–500
Platelet concentrate	30	5	–
Estimated blood loss (mL)	450		300–650
Preoperative laboratory results	Values		IQR
Hb (g/dL)	12.8		11.3–14.1
Alb (g/dL)	4.1		3.6–4.4
GFR (mL/min)	77.1		61–91.3
LDH (IU/L)	318		196–402
CRP (mg/L)	0.7		0.1–18.2
PLT (× 10^3^/μL)	221		178.3–270.8
Neutrophil (%)	60.7		53.7–69.5
Lymphocyte (%)	27.5		19.7–34.3

Values are presented as number (%) or median.

*CRP* C-reactive protein, *GFR* glomerular filtration rate, *IQR* interquartile range, *LDH* lactate dehydrogenase, *PLT* platelet.

### Common gastrointestinal tract complications

The overall complication rate was 56%, and 15% of the complications were observed within 30 days (105 patients) of surgery. The incidence of major and minor complications was 18% (129 patients) and 38% (273 patients), respectively. Among all categories, the gastrointestinal complication rate was 24% (173 patients), with 31% (47 patients) experiencing high-grade complications. The most common gastrointestinal complications encountered were prolonged POI (93 patients, 13%), small bowel obstruction (29 patients, 4%), nausea/vomiting (27 patients, 4%), and diarrhea (37 patients, 5%) (Table 3).

### Association between intravenous fluid amount, POI period, and length of hospital stay

The POI period was positively associated with crystalloid (γ = 0.227, P < 0.001) and total intravenous fluid volumes (γ = 0.242, P < 0.001), and not associated with colloid volumes. LOS was positively associated with crystalloid (γ = 0.208, P < 0.001), colloid (γ = 0.097, P = 0.010), and total intravenous fluid volumes (γ = 0.211, P < 0.001).

### Regression analysis

**Figure 1:**
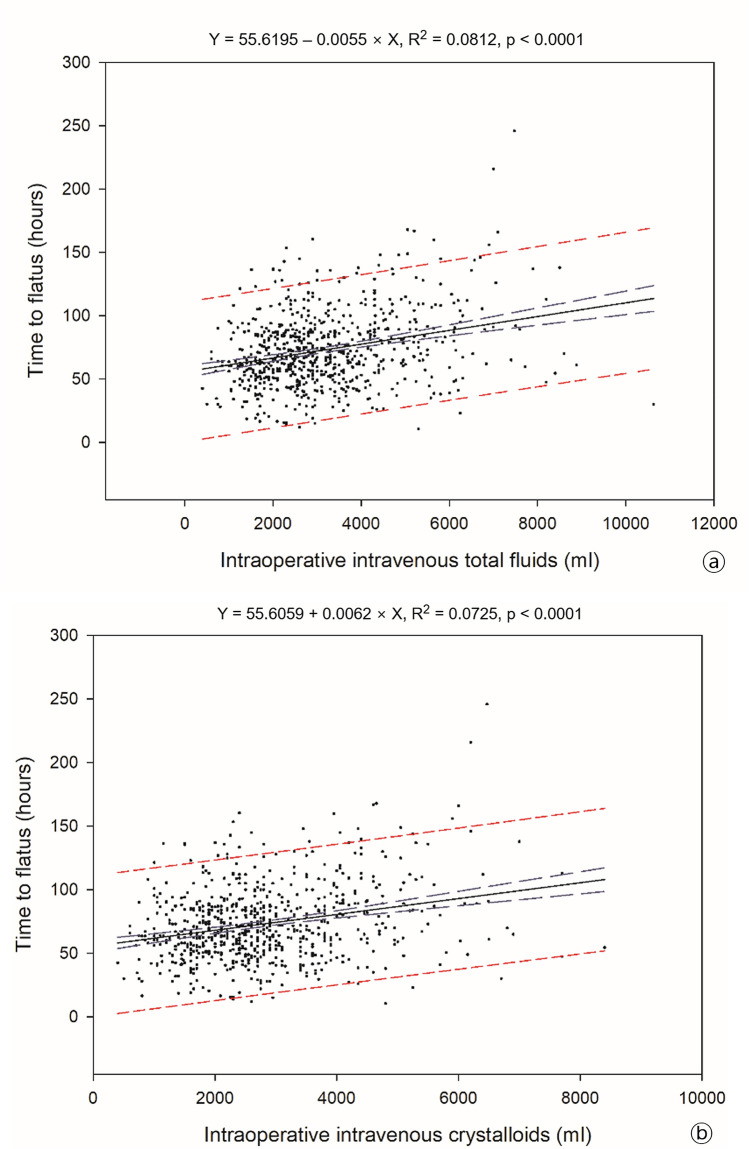
Linear regression analysis of the associations between intraoperative intravenous fluids and the postoperative ileus period as time to flatus; (**a**) total fluids, (**b**) crystalloid (blue long dash line—95% confidence band, red short dash line—95% prediction band).

**Figure 2:**
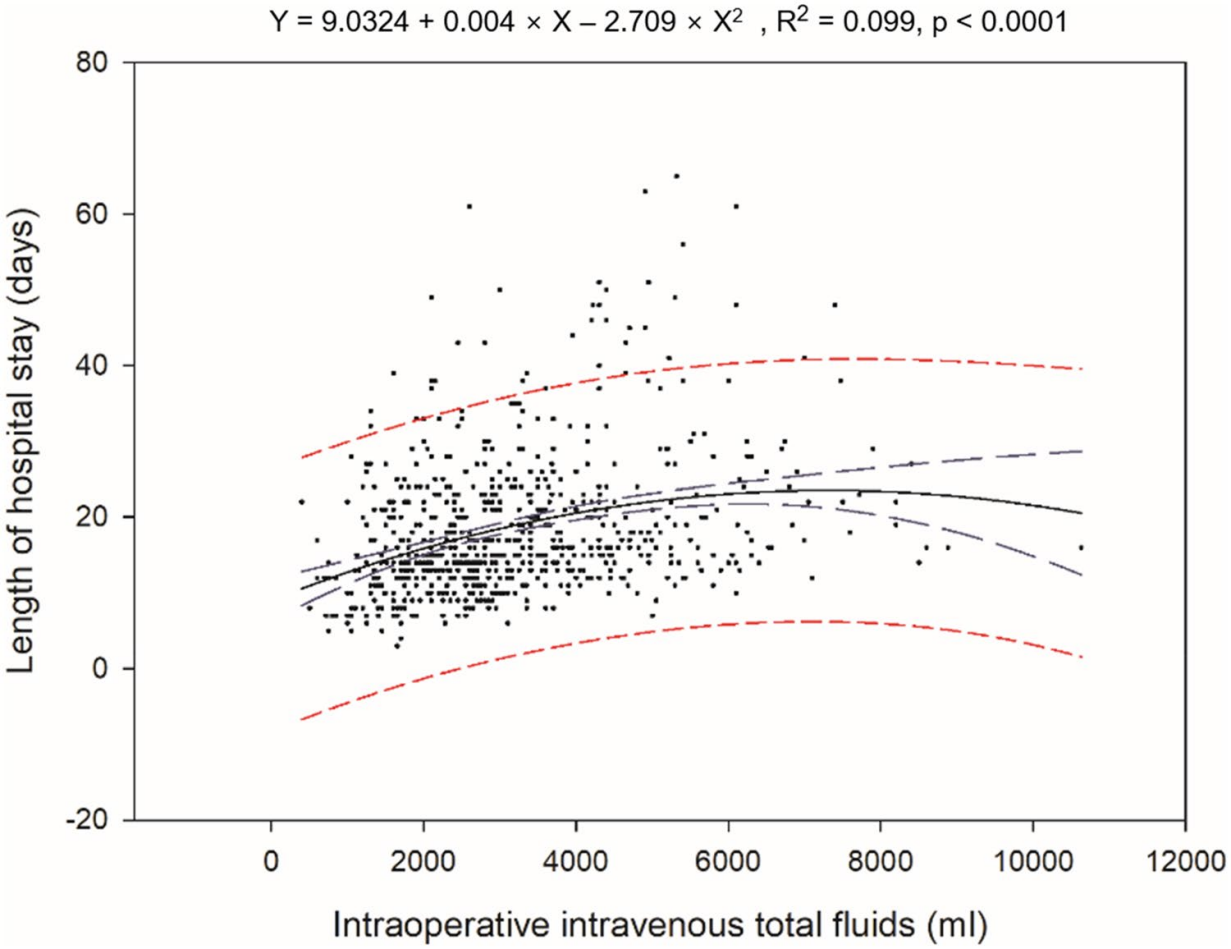
Regression curves on the analysis of the association between intraoperative intravenous total fluids and length of hospital stay.

On multivariate linear regression analyses, the total intraoperative intravenous volume and crystalloid volume were significantly positively associated with the time to flatus (total volume, P < 0.0001; crystalloid, P < 0.0001; Fig. 1a, b). In addition, the total intraoperative intravenous volume was the only significant factor for LOS (P < 0.0001; Fig. 2).

### Predictive factors of gastrointestinal complication and prolonged POI

On logistic regression analysis, the type of diversion technique was a significant predictor of gastrointestinal tract complications (odds ratio [OR], 1.985; 95% confidence interval [CI], 1.198–3.288; P = 0.008), while the fluid volume was not statistically significant. As for prolonged POI, the crystalloid amount (OR, 1.361; 95% CI, 1.133–1.641; P < 0.001) was statistically significant (Table 4). In addition, other clinical factors, such as operation time, age, sex, ASA score, PLND extent, and TNM stage (not mentioned in Table 4), did not show statistical significance (Table 4).

**Table 3:** Summary of the common gastrointestinal tract complications experienced.

	n = 718 no. (%)
Total complication rate	402 (56)
Within 30 days	105 (15)
Within 90 days	297 (41)
Major complication rate (Clavien-Dindo grades 3–5)	129 (18)
Minor complication rate (Clavien-Dindo grades 1–2)	273 (38)
Gastrointestinal complication rate	
Overall	173 (24)
Major complication rate (Clavien grades 3–5)	47 (31)
Prolonged postoperative ileus	93 (13)
Small bowel obstruction	29 (4)
Diarrhea	37 (5)
Others (hematochezia, stomach/duodenal ulcer)	9 (1)

**Table 4:** Logistic regression analysis of variables associated with gastrointestinal complication and paralytic ileus treated with robot-assisted radical cystectomy.

Variable	GI tract complication			Prolonged POI ^a^		
	OR	95% CI	P value	OR	95% CI	P value
BMI	0.995	0.893–1.109	0.929	0.301	0.09–1.03	0.066
BSA	0.229	0.023–2.313	0.212	0.987	0.94–1.03	0.542
Operation time	1.000	0.998–1.002	0.936	1.001	1.000–1.003	0.108
Diversion technique (OBS vs. Ileal conduit)	1.985	1.198–3.288	0.008*	0.071	0.02–0.25	0.132
Corporeal type (Intracorporeal vs. extracorporeal)	0.799	0.470–1.358	0.407	0.316	0.08–1.19	0.745
Crystalloid injection amount, mL	1.012	0.99–1.03	0.311	1.361	1.133–1.641	< 0.001**
Colloid injection amount, mL	1.004	0.997–1.010	0.925	1.001	1.00–1.003	0.068

*BMI* body mass index, *BSA* body surface area, CI confidence interval, *GI* gastrointestinal, *OBS* orthotopic bladder substitution, *OR* odds ratio, *RARC* robot-assisted radical cystectomy.

*P < 0.05, **P < 0.01.

^a^Definition: described in the Materials and Methods section.

## Discussion

The main results of this multi-institutional trial provide evidence for the association of POI period with the volume of intravenous fluid administered during RARC, a topic that has long been debated. The present study analyzed the impact of intraoperative fluid volume on the POI period in a cohort of 718 patients who underwent RARC, using regression analysis, on a large-scale, prospectively collected, KORARC database of patients. As hypothesized, we found that liberal intraoperative fluid volume was associated with prolonged POI and longer LOS, which implies that stringent fluid management could help decrease the postoperative recovery time after RARC.

The main strength of this study was that it specifically evaluated the independent impact of intraoperative fluid volume on the recovery of bowel function in the context of complex radical surgery, limiting the risk of bias due to several intraoperative confounding factors. On regression analyses, our results confirmed that the administration of less fluid produced small, but significant postoperative recovery advantages (R^2^ = 0.0812 for POI and 0.099 for LOS).

The goal of intraoperative fluid management is to create a “zero” fluid balance at the end of the surgery without complications. For physiological maintenance, enough fluid should be given to maintain the patient's weight prior to surgery, and for replacing fluid loss through urine, sweat, and other routes^24^. The volume of balanced crystalloid solution administered should not exceed 3 mL kg^-−1^ h^−1^, because during major abdominal surgery, only 0.5–1.0 mL kg^−1^ h^−1^ of water is lost due to evaporation (lower than originally thought)^25^. Clinically, bowel edema, which results in ileus, is the most common symptom occurring secondary to excessive fluid therapy during RARC, and tends to be more prominent under the care of inexperienced anesthesiologists. RARC is a relatively complex operation that takes approximately 6 h on average (the volume of crystalloid administered increases with the duration of surgery), and pneumoperitoneum and head-down positions make fluid therapy indices difficult to interpret. Several studies have found that perioperative fluid overload is associated with increased morbidity^26^, and it has been demonstrated that avoiding fluid overload leads to improved outcomes not only in urology, but also in major gastrointestinal surgery^27^. In the Multicenter Danish study, when fluid was restricted during surgery, postoperative complications were reduced by almost half regardless of the volume of fluid infusion before and after surgery^10^. Lobo et al.^28^ reported that fluid overload during elective surgery was related to increased time until the first passage of flatus or stool, increased gastric emptying time, and increased time to tolerance of solid food.

The reconstructive portion of the RARC for bowel resection and anastomosis is closely associated with POI. Marjanovic et al. demonstrated that hypervolemia increases intravascular hydrostatic pressure and causes damage to the endothelial glycocalyx, a mediator that induces fluid retention in the interstitial space^17,29,30^. In their animal model, a distinct submucosal pale band was found in the hypervolemia group, and a narrow and dense submucosal layer was found in the volume-restriction group. In addition, the volume of crystalloid infusion during surgery was reported to have a significant effect on the structural and functional stability of the intestinal anastomosis in the early postoperative period.

However, there are a few issues in our findings that need to be addressed. First, in our results, colloids, unlike crystalloids, were not a risk factor for POI. Some studies have found a greater influence of colloids on the fluid resuscitation of critically ill patients compared with crystalloids; but studying how colloids might impact POI is largely a novel area of research^31,32^. Crystalloid have a hemodilution effect, while colloids have the ability to maintain osmotic pressure in the vasculature; hence, even with the same volume, colloids are expected to induce lesser bowel edema.

We previously investigated whether intracorporeal urinary diversion (ICUD) has more clinical benefits than extracorporeal urinary diversion (ECUD)^33^. Our study showed significantly better results for ICUD compared to ECUD in terms of the overall and gastrointestinal complication rates, which is consistent with the International Robotic Cystectomy Consortium data^34^. The duration for which the peritoneum is exposed to the external environment is proportional to intestinal inflammation and oxidative stress response, and these factors could cause the delayed restoration of bowel function^35^. However, in our study, the corporeal type did not significantly affect the occurrence of gastrointestinal complications (P = 0.407) or prolonged POI (P = 0.745). This is attributed to the following reasons: The anesthesiologist of each operation was different, which resulted in differing strategies of fluid therapy administration. Moreover, our data did not reflect the clinical experience of surgeons, which means that a few outcomes of surgeons who had not overcome the learning curve yet were included.

Our study has several limitations. First, further fluid management-related measures, such as urine output, were not measured. However, owing to the surgical principles of RARC that include bilateral ureter clamping in the early stage of surgery, the impact might be insignificant and can be a confounding factor considering that it is difficult to accurately account for. Certainly, it should be acknowledged that various surgical situations warrant varying interventions and different surgeons/anesthesiologists, potentially limiting the generalizability of these results in centers with different principles of perioperative care.

In conclusion, intravenous fluid administration during surgery is an easily modifiable part of RARC, and intense collaboration between urologists and anesthesiologists is necessary to encourage optimal gastrointestinal recovery. Within its limitations, this study shows that an increased volume of fluid received during RARC may impair return to normal bowel function. To maximize the advantage of RARC as a minimally invasive operation, careful monitoring is required to control the amount of fluid infusion during surgery and to avoid fluid overload.
